# Microsurgical and illustrative anatomy of the cavernous sinus, middle fossa, and paraclival triangles: a straightforward, comprehensive review

**DOI:** 10.1007/s00276-023-03105-y

**Published:** 2023-02-28

**Authors:** Víctor Ramzes Chavez-Herrera, Álvaro Campero, Daniel Ballesteros-Herrera, Bayron Alexander Sandoval-Bonilla, Cristian Alberto Perez-Carrillo, Diego Tonathiu Soto-Rubio, Eduardo Javier Valladares-Pérez, Pedro Adrián González-Zavala, Luis Alfonso Castillejo-Adalid, Job Jesús Rodríguez-Hernández

**Affiliations:** 1 Department of Neurosurgery, Hospital de Especialidades, Instituto Mexicano del Seguro Social Centro Médico Nacional Siglo XXI, Ciudad de México, Mexico; 2 Department of Neurosurgery, Padilla Hospital, Tucumán, Argentina; 3 Department of Neurosurgery, Instituto Nacional de Neurología y Neurocirugia, Manuel Velasco Suarez, Ciudad de México, Mexico

**Keywords:** Middle fossa, Cavernous sinus, Paraclival, Anatomy, Triangles, Review

## Abstract

The middle fossa, cavernous sinus, and paraclival triangles consist of ten triangles. Their use in a surgical approach is vast; most are used as landmarks to access and identify other structures of surgical interest. Multiple labels, borders, and contents mentioned by different authors make understanding and reproduction challenging and confusing. This study aims to organize and clarify recent or most relevant publications and disclose our portrayal of the ten triangles using cadaveric dissection and simple and practical figures. Four middle fossa triangles, four cavernous sinus triangles, and two paraclival triangles were dissected and delineated in a cadaveric specimen. Drawings were simplified to eliminate confusion and evaluate the triangles effortlessly. Similarities and differences in triangle names, border limits, and contents are described in a precise form. The recognition of triangle landmarks allows for treating pathologies in a frequently distorted anatomy or challenging to access structure. That is why an accurate knowledge of the surgical anatomy should be mastered, and a safe approach should be accomplished.

## Introduction

For years, there has been confusion when determining the name and border descriptions of the cavernous sinus and middle fossa triangles. Various authors have mentioned multiple names, border defining limits, and contents that cause confusion and learning distrust. For this reason, we present a brief revision of history, triangle differences, and similarities described in different publications. Finally, we explain a knowledgeable description using our point of view concerning each triangle using cadaveric dissection and simple figures.

Multiple publications mention the practical use of the triangles. Escudeiro et al. describe the utilization of the Parkinson´s triangle to access a cavernous sinus hemangioma [7]. Kusumi et al. used an extra-dural middle fossa approach to remove a schwannoma in the Glasscock triangle [21]. Ferrareze et al. performed an endoscopic endo-nasal approach through the oculomotor triangle to remove an extended pituitary tumor in the para-peduncular space [8]. Watanabe et al. access the anterior temporal fossa to the paranasal sinuses and nasal cavities through the anterolateral and anteromedial triangles in a microscopic and endoscopic approach [30]. For this reason, recognizing and comprehending the ten triangles´ anatomy are critical to a safe and successful surgical approach.

## Materials and methods

We used a cadaveric specimen injected with red silicon for arteries and blue for veins. Specimen preserved in a 70% alcohol solution and refrigerated. A head holder was used to keep the head in the correct position. A Pico microscope (Zeiss) was used for intracranial visualization. Midas drill (Medtronic) was used to perform a cranio-orbital approach. Microsurgical instruments, bipolar, and 11 scalpels were used. Dissection was documented step by step with a DCLR camera Sony A6300. Additional processing was done with Photoshop (Adobe) and Helicon Focus. Dissection was made at the laboratory of the Centre Hospitalier Universitaire Vaudois.

We searched PubMed (http://www.ncbi.nlm.nih.gov/pubmed/) for “cavernous sinus triangles,” “middle fossa triangles,” “paraclival triangles,” “oculomotor triangle,” “clinoidal triangle,” supratrochlear triangle,” “infratrochlear triangle,” “anteromedial triangle,” “anterolateral triangle,” “posteromedial triangle,” “posterolateral triangle,” inferomedial triangle” and “inferolateral triangle.” The most relevant or recently published articles were used. Also, remarkable book literature was considered. The name, borders, and contents were analyzed (Table 1).

**Table 1:** Triangle description by different authors

Name	Other names	Borders	Content
Oculomotor triangle	Hakuba´s triangle	1.Anterior petroclinoid dural fold	Oculomotor Nerve
Dolenc [5]	Drazin et al. [6]	2. Posterior petroclinoid dural fold	ICA horizontal segment
		3. Interclinoid dural fold	Drazin et al. [6]
	Medial triangle	Drazin et al. [6]	
	Drazin et al. [6]		
		1. Medial border: A line between the anterior and posterior clinoid process	
		2. Lateral border: the fold of the dura between the anterior clinoid process and petrous apex	The site where the oculomotor nerve enters the roof of the cavernous sinus
		3. Base: the fold of the dura from the posterior clinoid process to the petrous apex	Distal intra-cavernous carotid artery
		Isolan et al. [14]	Isolan et al. [15]
		1. Anterior petroclinoid fold: petrous apex to the anterior clinoid process	Cavernous sinus
		2. Posterior petroclinoid fold: petrous apex to the posterior clinoid process	Gallardo et al. [10]
		3. Interclinoid fold: anterior and posterior clinoid process	
		Gallardo et al. [10]	
Clinoidal triangle	Dolenc´s triangle	1. Optic nerve	Clinoidal internal carotid artery
Isolan et al. [14]	Fujimoto et al. [9]	2. Oculomotor nerve	Anterior clinoid process
		3. Tentorial edge (a line between the dural entry point of the third cranial nerve and the optic nerve)	Drazin et al. [6]
	Anteromedial triangle	Drazin et al. [6]	
	Dolenc [5]		Anteriorly: Optic strut
		1. Medial border: optic nerve	Middle: Subclinoidal segment of the ICA
	Anterior triangle	2. Lateral border: third cranial nerve from the entry point in the sinus roof to the point just before entering the superior orbital fissure	Posteriorly: the roof of cavernous sinus (after drilling de anterior clinoid process)
	Gallardo et al. [10]	3. Base: the dura extending between the posterior limits of the medial and lateral border	Isolan et al. [14]
		Isolan et al. [14]	
Supratrochlear triangle	Paramedian triangle	1. Medially: oculomotor nerve	Meningohypophyseal trunk, the inferolateral trunk and less commonly the medial loop of the ICA
Isolan et al. [14]	Goel [11]	2. Laterally: trochlear nerve	Drazin et al. [6]
		3. Inferiorly: tentorial edge (the dura extending between the dural entry points of the third and the fourth cranial nerves)	
		Watanabe et al. [29]	
	Paramedial triangle		
	Dolenc [5]	1. Oculomotor nerve	
		2. Trochlear nerve	
	Superior	3. Tentorial edge	
	Fukushima	Drazin et al. [6]	
	Wanibuchi [28]		
Infratrochlear triangle	Parkinson´s triangle	1. Medially: trochlear nerve	ICA (cavernous) and the abducens nerve
Isolan et al. [14]	Dolenc [5]	2. Laterally: ophthalmic division of the trigeminal nerve	
		3. Base: tentorial edge of these two nerves	Drazin et al. [6]
	Superolateral triangle	Watanabe et al. [29]	
	Watanabe et al. [29]	1. Trochlear nerve	Posterior–superior, anterior–inferior, and lateral venous spaces and lateral surface of the C5 and C6
		2. Ophthalmic division (V1)	Watanabe et al. [29]
		3. Tentorial edge	
		Drazin et al. [6]	Origin of the meningohypophyseal trunk
			Peltier et al. [24]
		1. Superiorly: lower margin of the trochlear nerve	
		2. Inferiorly: upper rim of the ophthalmic nerve and of the trigeminal ganglion	
		3. Posterior: slope of the dorsum sellae and clivus	
		Kayalioglu et al. [17]	
Anteromedial triangle	Mullan´s triangle	1. Lower margin of ophthalmic nerve	Sphenoid sinus
Conti et al. [3]	Hakuba et al. [13]	2. Upper margin of maxillary nerve	Granger et al. [12]
		3. Line connecting superior orbital fissure and foramen rotundum	
		Granger et al. [12]	Superior orbital fissure artery
	Anterolateral triangle		Conti et al. [3]
	Dolenc [5]	1. Upper margin of maxillary nerve	
		2. Line connecting the ophthalmic nerve at superior orbital fissure and the maxillary nerve at foramen rotundum	Pituitary gland
		Rhoton [26]	Watanabe et al. [29]
Anterolateral triangle	Lateral triangle	1. Lower Surface of maxillary nerve	Lateral wing of the sphenoid sinus
Kobayashi [19]	Dolenc [5]	2. Upper surface of the mandibular nerve	Rhoton [26]
		3. Line connecting the foramen ovale and rotundum	
	Far Lateral	Rhoton [26]	Lateral wing of the sphenoid sinus
	Day et al. [4]		Vidian nerve
		1. Medial border: Lower surface of the maxillary nerve,	Pterygoid region
	Lateralmost	2. Lateral border: upper surface of the mandibular nerve	Granger et al. [12]
	QuinonesHinojosa [25]	3. Base: a line connecting the foramen ovale and rotundum	
		Watanabe et al. [29]	
		1. Posterior border: maxillary division of the trigeminal nerve	
		2. Anterior border: mandibular division of the trigeminal nerve	
		3. Line connecting the foramen rotundum to the foramen ovale	
		Granger et al. [12]	
Posterolateral Triangle	Glasscock	1. Medial border: Line between where the GSPN crosses under V3 and the foramen spinosum	Posterior and lateral loops of ICA
Dolenc [5]	Dolenc [5]	2. Lateral border: Line between the foramen spinosum and geniculate ganglion	Greater and lesser petrosal nerves
		3. Base: GSPN	Tensor tympani muscle
	Paullus	Isolan et al. [14]	Eustachian tube
	Wanibuchi [28]		Middle meningeal artery
		1. Medial border: a line between the points on the lateral surface of the mandibular nerve where the greater petrosal nerve crosses to the foramen spinosum	Infratemporal fossa
		2. Lateral border: line between the foramen spinosum and the center of the geniculate ganglion	Isolan et al. [14]
		3. Base: medial margin of the greater petrosal nerve	
		Watanabe et al. [29]	
Posteromedial Triangle	Kawase	1. Medial border: GSPN	Posterior cavernous sinus
Dolenc [5]	Dolenc [5]	2. Lateral border: Line between where the GSPN crosses under V3 and the petrous apex	Entry point to the posterior fossa (anterior petrosectomy)
		3. Base: Line between the crest of the petrous apex to the geniculate ganglion	Lateral apex: Cochlea, anterior wall of the IAC
	Kawase-Shiobara	Watanabe et al. [29]	Anterior margin: Petrous carotid
	Kanzaki		Medial margin: Clivus and inferior petrosal sinus
	Wanibuchi [28]		Isolan et al. [14]
		1. Anterior border: V3	
		2. Posterior border: Arcuate Eminence	
		3. Lateral border: GSPN	
		4. Medial border: Petrous ridge	
		Isolan et al. [14]	
Paraclival	None	1. Posterior clinoid process	Dorello’s canal and Gruber’s ligament
Inferomedial		2. Dural entrance of the trochlear nerve	Drazin et al. [6]
Dolenc [5]		3. Dural entrance of the abducens nerve	
		Isolan et al. [14]	Dura forming the posterior wall of the cavernous sinus, the abducens nerve, the petrosphenoidal (Grüber’s) ligament,
			The posterior genu of the internal carotid artery’s intracavernous segment
		1. Posterior clinoid process	Dorsal meningeal artery
		2. Dural entry of the Trochlear nerve	Wysiadecki et al. [32]
		3. Dural entry of the abducens nerve	
		Wysiadecki et al. [32]	The abducens nerve
			the posterior genu of the ICA
		1. Medial: posterior clinoid process	the dorsal meningeal artery
		2. Supero-lateral: Dural entry of the Trochlear nerve	the basilar venous plexus
		3. Inferolateral: Dural entry of the abducens n	the posterior petroclinoid fold
		Dolenc [5]	Isolan et al. [14]
Paraclival	None	1. Medially: by a line between the dural entrance of the trochlear nerve into the tentorium cerebelli to the dural entry of the abducens nerve	Porus trigemini (the Meckel’s cave)
Inferolateral		2. Laterally by a line between the dural entry point of the abducens nerve and the petrosal vein	Drazin et al. [6]
Dolenc [5]		3. Petrous apex	
		Drazin et al. [6]	
		1. Line between the entry point of CN IV at the tentorium and CN VI at Dorello’s canal	Porus trigeminus
		2. Line between entry point of CN VI at Dorello’s canal and the superior petrosal vein at the superior petrosal sinus	The entrance of the sixth nerve in Dorello’s canal
		3. Line between entry point of CN IV at the tentorium and the superior petrosal vein at the superior petrosal sinus	The fourth cranial nerve entrance along the incisura into the lateral wall of the cavernous sinus
		Isolan et al. [14]	Isolan et al. [14]
		1. Anterior border: line connecting the dural entry point of the trochlear nerve to the dural entry point of the abducens nerve	
		2. Posterior border: line connecting the dural entry point of the abducens nerve to the drainage site of the petrosal vein into the superior sagittal sinus	
		3. Superior border: line connecting the dural entry point of the trochlear nerve to the petrosal vein	
		Kimball et al. [18]	

According to our cadaveric specimens, digital drawings of the ten triangles were optimized and simplified, eliminating distracting surroundings. The digital application platform “Procreate” was used in all drawings.

## Results

### Oculomotor triangle

**Fig. 1:**
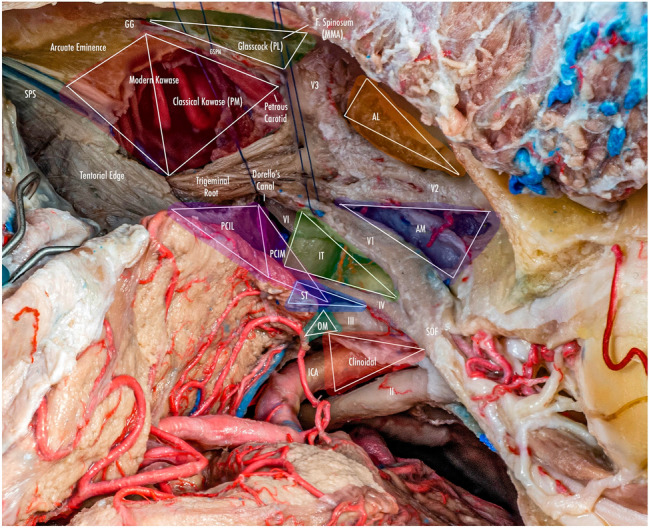
Anterolateral aspect of the middle cranial fossa depicting the triangles formed in this region. The roof and lateral aspect of the orbit have been drilled. The Sylvian fissure is shown splittled. The retractor is over the temporal lobe. From medial to lateral, the clinoidal triangle has been exposed after an anterior clinoidectomy has been done. It is between the optic and the oculomotor nerves and posteriorly bordered by the tentorial edge (Not shown). The oculomotor triangle (OM) is the site where the oculomotor nerve becomes extradural by entering the upper portion of the lateral wall of the cavernous sinus. Its margins are the anterior petroclinoial dural fold extending from the ACP to the petrous apex and the posterior petroclinoidal dural folds extending from the posterior clinoidal process to the petrous apex and medially by the interclinoidal dural fold. The supratrochlear triangle (ST), the space between the oculomotor and the trochlear nerves, has a posterior border drawn by a line at the dural entry point of these two nerves. The infratrochlear triangle (IT/Parkinson’s triangle) is lateral to the oculomotor and medial to the trochlear nerve. Its posterior border is the tentorial edge between these two nerves. The anteromedial triangle’s (AM/Mullan’s triangle) boundaries are the ophthalmic division of the trigeminal nerve medially and the maxillary division laterally. Its base is formed by a line connecting the superior orbital fissure to the foramen rotundum over the bony middle cranial fossa wall. The anterolateral triangle (AT) is formed medially by the maxillary division of the trigeminal nerve and laterally by the mandibular division (V3). The base is formed by a line connecting the foramen rotundum and the foramen ovale. Posteriorly over the middle cranial fossa, the Posteromedial and the posterolateral triangles can be found. The first of these two, the Posteromedial Middle Fosa Triangle (AKA Kawase’s triangle), is bordered laterally by the medial margin of the greater superficial petrosal nerve (GSPN). The petrous ridge is found medially. Anteriorly its boundary is the mandibular division of the trigeminus and laterally by V3. Posteriorly, the limit is the arcuate eminence. The posterolateral middle fossa triangle (Glasscock) is located laterally to the line where the GSPN crosses under V3 and the foramen spinosum. Its lateral border is a line between the foramen spinosum and the geniculate ganglion. Its base is GSPN. The paraclival triangles are the Inferomedial and Inferolateral triangles (PCIM and PCIL). The inferomedial triangle contains the dura forming the posterior wall of the cavernous sinus. It is delimited medially by a line extending from the posterior clinoid process to the dural entry of the abducens nerve. Its lateral border is a line extending from the posterior clinoid process to the dural entry of the trochlear nerve. Its base is the line extending from the dural entry of the abducens nerve and the trochlear nerve. Over the posterior surface of the clivus and the temporal bone, we can find the Inferolateral triangle (PCIL). Its anterior border is a line extending from the dural entry of the abducens nerve and the trochlear nerve's dural entry. Its lateral border is a line extending from the entrance of the trochlear nerve and the petrosal vein. Its posterior border is a line extending from the dural entry of the abducens nerve to the petrosal vein

**Fig. 2:**
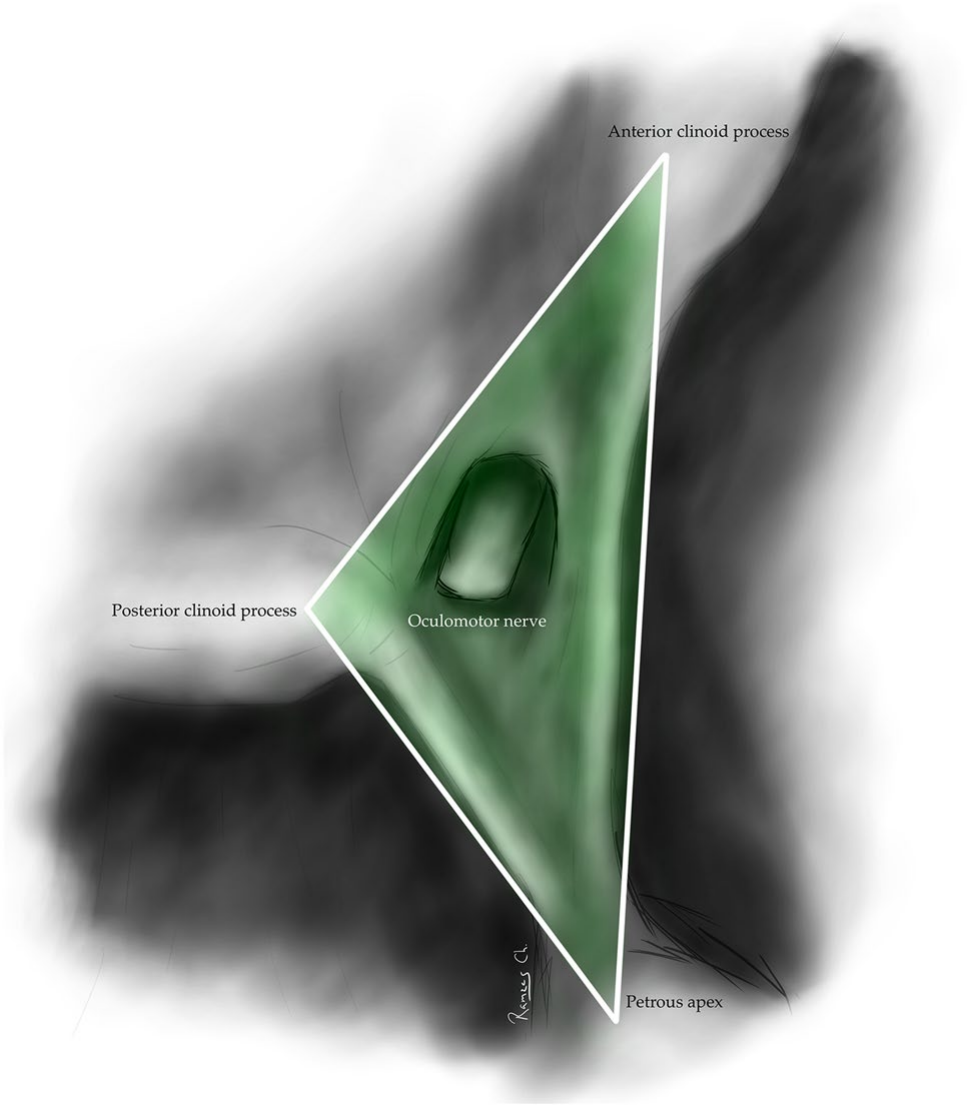
Oculomotor triangle: It is bordered anteriorly and posteriorly by the dural folds attached to the petrous apex and connected to the anterior clinoid process and posterior clinoid process (Anterior Petroclinoidal and Posterior petroclinoidal dural folds), respectively. Medially, its limit is the interclinoidal dural fold

The oculomotor triangle (Hakuba´s triangle and medial triangle) is delimited by three dural folds forming the medial or interclinoid border, lateral or anterior petroclinoid border, and posterior, base, or posterior petroclinoid border. In addition to surrounding the entry point of the third cranial nerve to the roof of the cavernous sinus, it contains the horizontal portion of the intra-cavernous segment of the internal carotid artery (ICA) [5, 6, 10, 14] (Figs. 1, 2, 4) (Table 1).

### Clinoid triangle

**Fig. 3:**
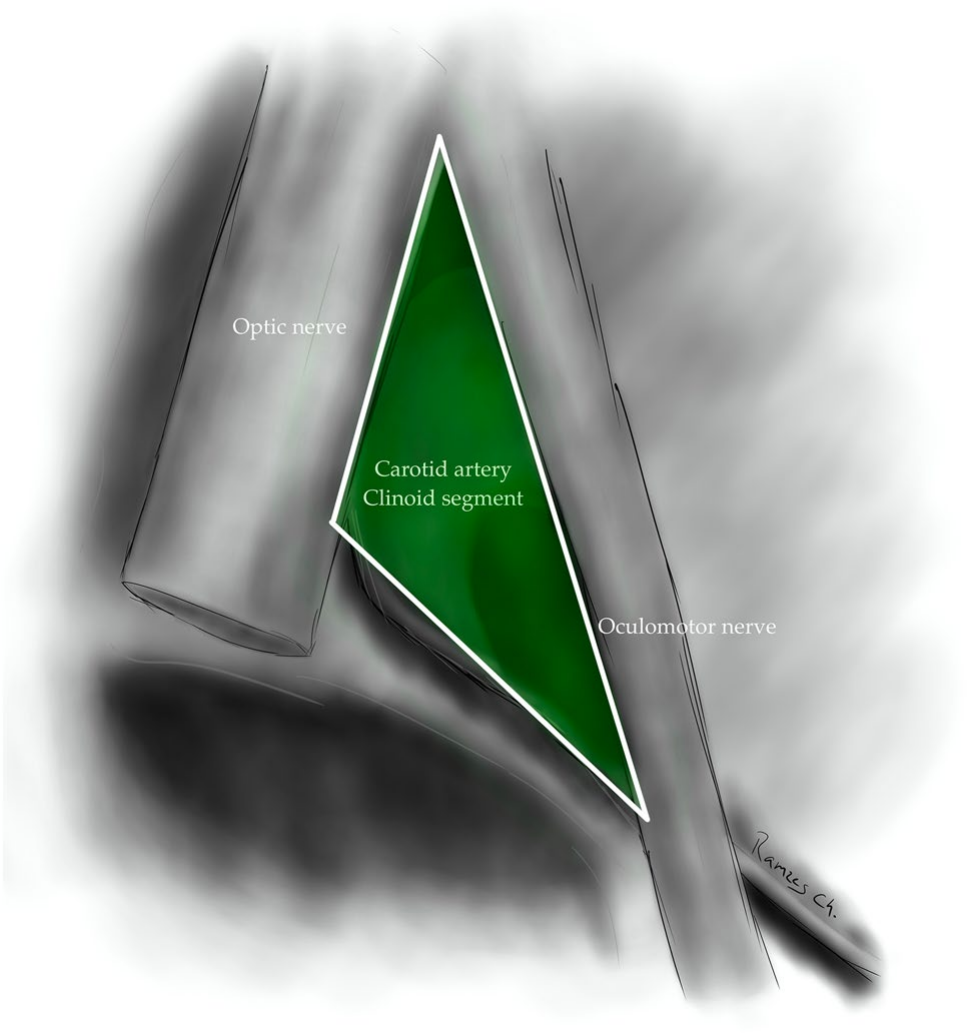
Clinoidal triangle (Dolenc’s, Anteromedial triangle); Bordered on its lateral side by the oculomotor nerve and limited medially by the optic nerve, posteriorly limited by the tentorial edge

**Fig. 4:**
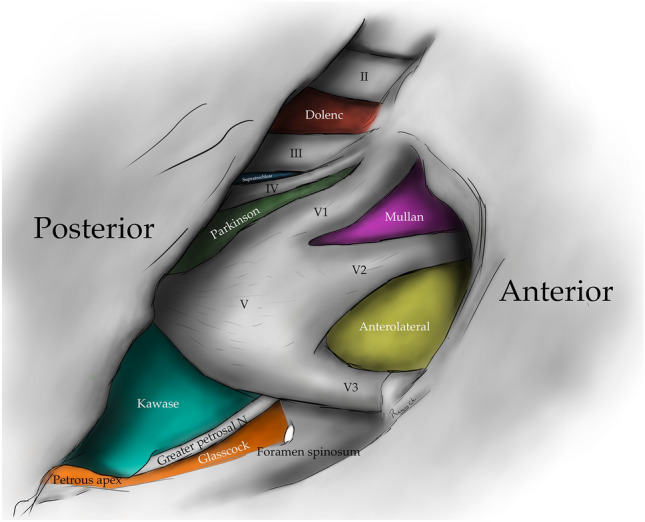
Drawing depicting the disposition of the triangles on the lateral wall of the cavernous sinus and middle cranial fossa

**Fig. 5:**
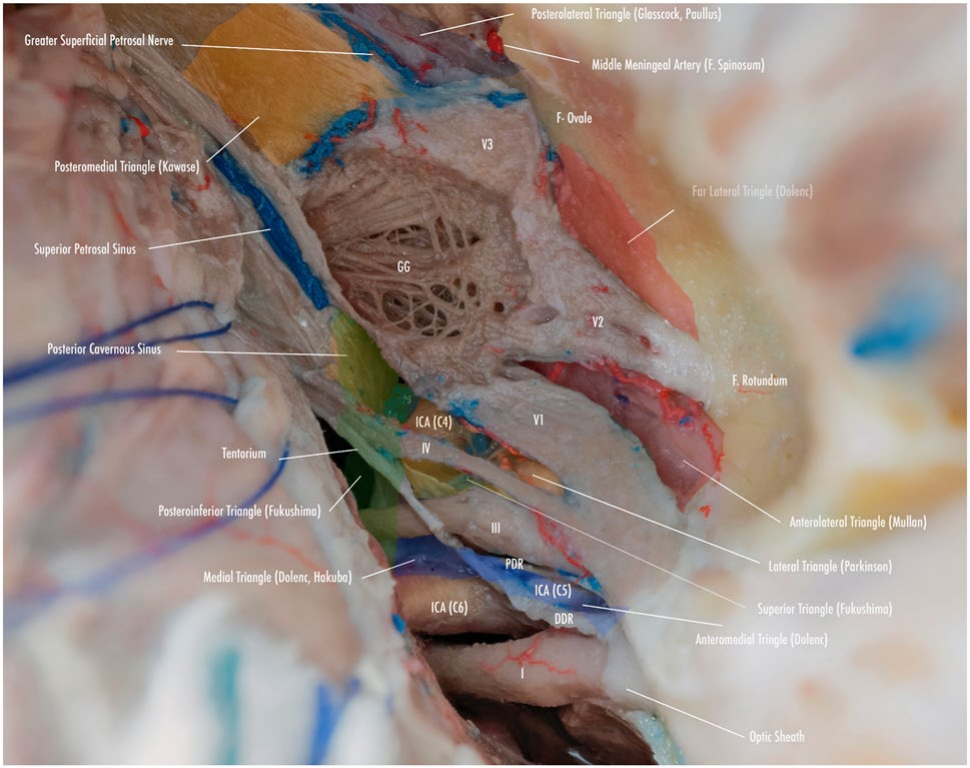
Anatomical dissection depicting the disposition of the triangles on the lateral wall of the cavernous sinus and middle cranial fossa

The clinoid triangle (Dolenc´s triangle, anteromedial triangle, and anterior triangle) is bounded on its medial border by the optic nerve, the lateral border by a line from the point of entry of the third cranial nerve in the roof of the cavernous sinus to its point of entry in the superior orbital fissure, and the posterior border, corresponding to a line joining the posterior limits of the medial and lateral borders. To visualize this triangle fully, it is necessary to drill the anterior clinoid process. It contains in its anterior portion the optic strut, in its medial portion the clinoid segment of the ICA, and in its posterior segment the roof of the cavernous sinus. [5, 6, 9, 10, 15] (Figs. 1, 2, 3, 4, 5) (Table 1).

### Supratrochlear triangle

**Fig. 6:**
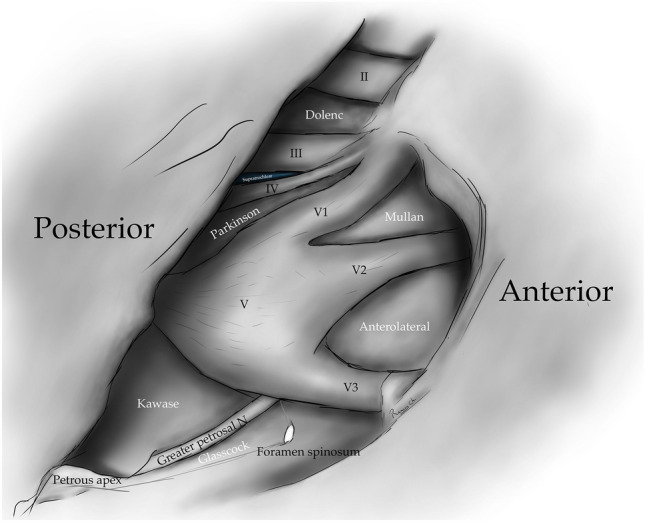
Supratrochlear triangle: Space bordered medially by the oculomotor nerve, laterally by the trochlear nerve. Its posterior border is the tentorial edge delimited by the dural entry point of these two nerves

The supratrochlear triangle (para-median triangle, para-medial triangle, superior triangle, and Fukushima´s triangle) corresponds to the space between the oculomotor and trochlear nerves at their medial and lateral borders, respectively, forming the posterior border with a line at the dural entry point of these nerves. Through this triangle, we can find the posterior curvature of the intra-cavernous segment of the ICA and, in some cases, the exit of the meningohypophyseal trunk, the inferolateral trunk, and, less frequently, the medial curve of the ICA. [5, 6, 11, 14, 28] (Figs. 4, 5, 6) (Table 1).

### Infratrochlear triangle

**Fig. 7:**
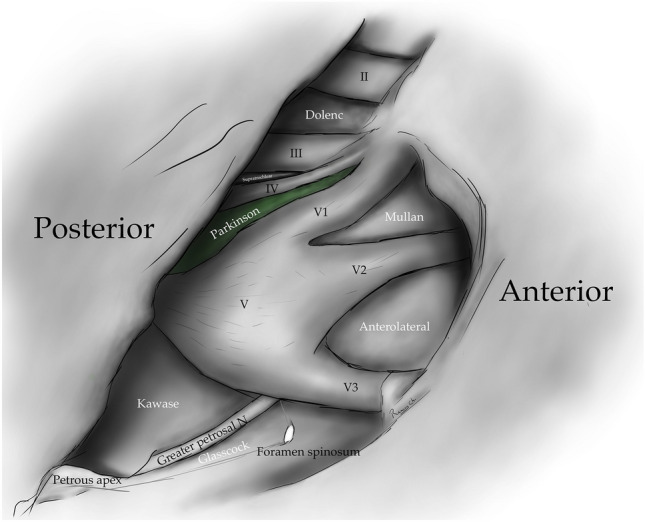
Infratrochlear triangle (Parkinson’s triangle) Over the lateral wall of the cavernous sinus. This space is bordered medially by the trochlear nerve and laterally by the ophthalmic division of the trigeminal nerve. The base of this triangle is drawn by the tentorial between these two nerves

The infra-trochlear triangle (Parkinson's triangle, supero-lateral triangle) is bounded medially by the trochlear nerve, laterally by the ophthalmic division of the trigeminal nerve, and posteriorly by a line joining the posterior limit of the medial and lateral borders. It generally contains the origin of the meningohypophyseal trunk and the intra-cavernous portion of the sixth cranial nerve [4–6, 14, 17, 24, 29] (Figs. 1, 4, 5, 7) (Table 1).

### Anteromedial triangle

**Fig. 8:**
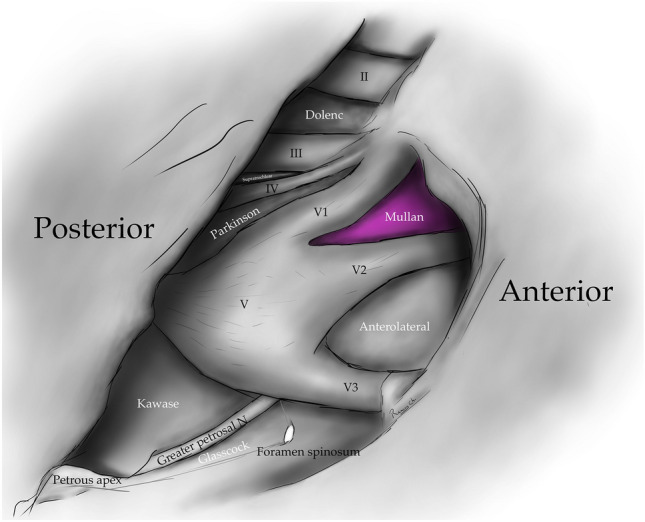
Anteromedial triangle (Mullan’s triangle); Its boundaries are the ophthalmic division of the trigeminal nerve medially and the maxillary division of the trigeminal nerve laterally. This space’s base is a line that connects the superior orbital fissure to the foramen rotundum over the bony middle cranial fossa

The anteromedial triangle’s (Mullan´s triangle and anterolateral) boundaries are formed by the ophthalmic division of the trigeminal nerve medially and the maxillary division of the trigeminal nerve laterally. The triangle base consists of the anterolateral wall of the bony middle cranial fossa formed by a line connecting the superior orbital fissure to the foramen rotundum. This corridor is well suited for exposing several important structures, including the superior orbital vein, sixth cranial nerve, sphenoid sinus, and ophthalmic vein. Further dissection within Mullan’s space allows for access to carotid-cavernous fistulas [3, 5, 12, 13, 26, 29] (Figs. 1, 4, 5, 8) (Table 1).

### Anterolateral triangle

**Fig. 9:**
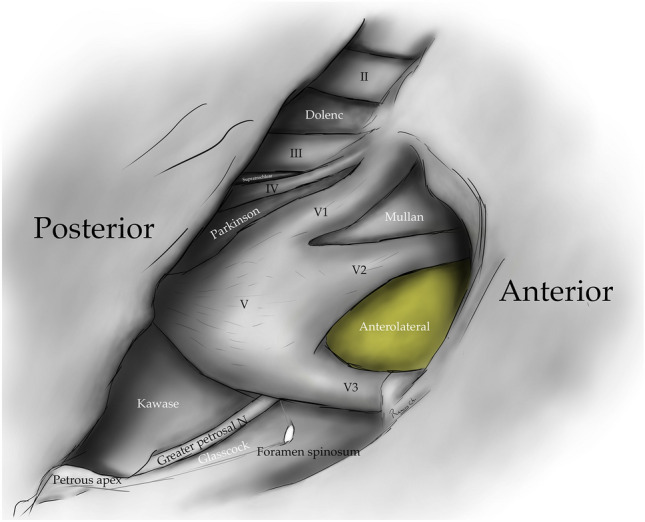
Anterolateral triangle; The lower margin of the maxillary nerve constitutes its medial border. The upper surface of the mandibular nerve is the lateral border. Anteriorly its base is a line between the foramen ovale and foramen rotundum

The anterolateral triangle (lateral triangle, far lateral triangle, lateral-most triangle) is formed medially by the maxillary division and laterally by the mandibular division of the trigeminal nerve. The base is identified via a line connecting the foramen rotundum and foramen ovale. The contents are the lateral wing of the sphenoid sinus, the Vidian nerve, and the pterygoid region. Far antero-inferior, the maxillary sinus can be exposed, and posteriorly, the infratemporal Eustachian tube can be exposed under the lateral and medial pterygoid muscles. This space exposes the lateral sphenoid wing, sphenoidal emissary vein, and cavernous-pterygoid venous anastomosis [4, 5, 12, 19, 25, 26, 29] (Figs. 1, 4, 5, 9) (Table 1).

### Posterolateral triangle

**Fig. 10:**
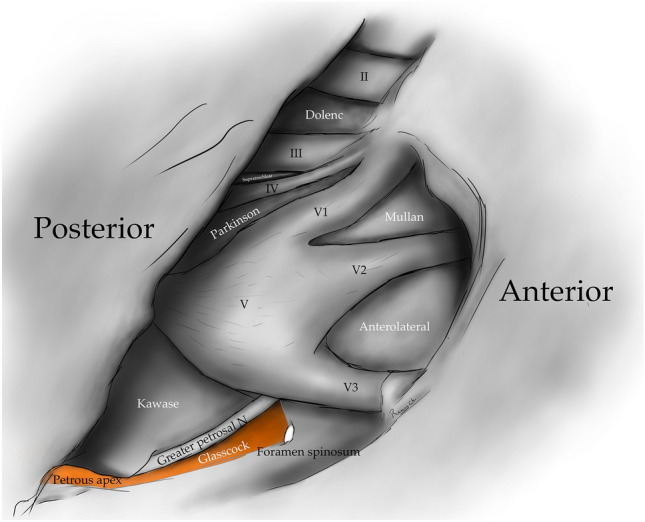
Posterolateral triangle (Glasscock, Paullus); Bordered medially by a line drawn between the point where the greater superficial petrosal nerve (GSPN) crosses under the mandibular division of the trigeminal nerve (V3) and the foramen spinosum, laterally; by a line drawn between the foramen spinosum and the geniculate ganglion. Its base is the GSPN

The posterolateral triangle (Glasscock´s triangle and Paullus´s triangle) is formed by the anteromedial side of the lateral surface of the mandibular nerve distal to the point at which the greater superior petrosal nerve (GSPN) crosses below the lateral surface of the trigeminal nerve. The anterior margin of the GSPN forms the posterolateral side. It opens laterally to encompass the floor of the middle cranial fossa between these two structures [1]. It contains the posterior and lateral loops of the ICA in its petrous segment, greater and lesser petrosal nerves, tensor tympani muscle, Eustachian tube, and middle meningeal artery that passes through the foramen spinosum. Opening the floor of this triangle exposes the infratemporal fossa [5, 14, 28, 29] (Figs. 1, 4, 5, 10) (Table 1).

### Posteromedial triangle

**Fig. 11:**
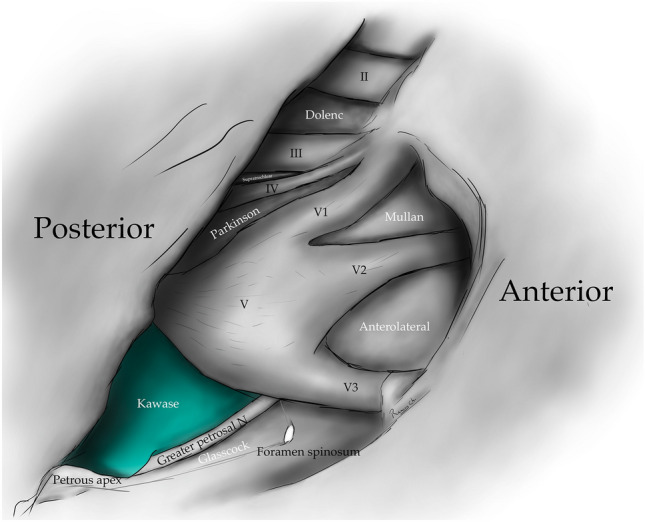
Posteromedial triangle (Kawase, Kawase-Shiobara, Kanzaki); recent descriptions mentioned a quadrilateral. Its limits are; Laterally, the medial margin of the greater superficial petrosal nerve. Medially; the petrous ridge, anteriorly the mandibular (V3) division of the trigeminal nerve. Posteriorly, the arcuate eminence

The posteromedial triangle (Kawase´s triangle, Kawase-Shiobara´s triangle, and Kanzaki´s triangle) was first described by Kawase [15, 16]. This triangle consists of a line between the hiatus fallopii and the dural ostium of the Meckel’s cave. Its posterior border is a line between the posterior border of the mandibular nerve and the center of the geniculate ganglion 15. Several structures surround it; at its lateral apex are the cochlea and anterior wall of the internal auditory canal (IAC), its anterior margin, the petrous carotid, and its medial margin, the clivus, and inferior petrosal sinus [16]. It contains the posterior cavernous sinus and the entry point to the posterior fossa exposed by performing an anterior petrosectomy. [5, 14, 28, 29] (Figs. 1, 4, 5, 11) (Table 1).

### Inferomedial paraclival triangle

**Fig. 12:**
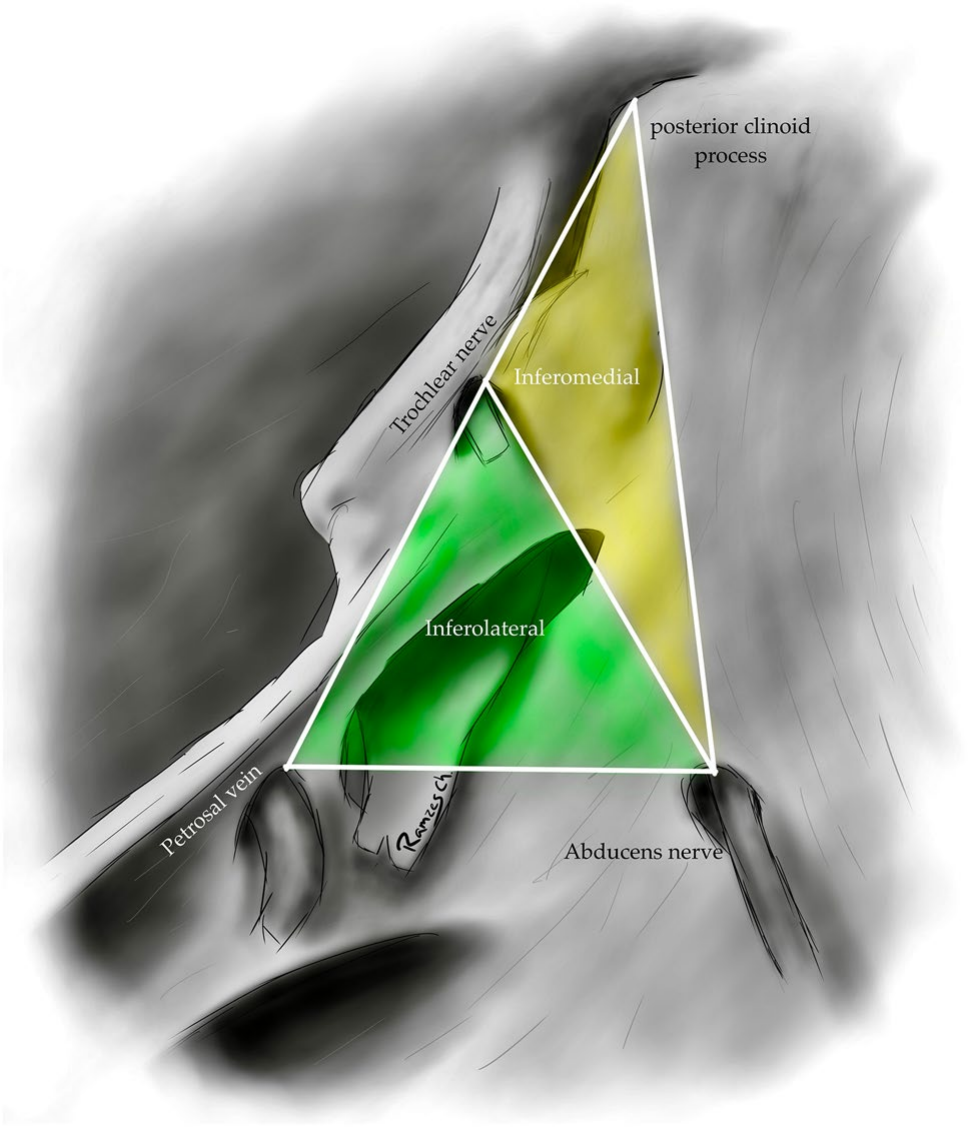
Image depicting the paraclival triangles; Inferomedial and inferolateral. The inferomedial triangle is delimited by the posterior clinoid process, the trochlear nerve's dural entrance, and the abducens nerve's dural entrance. The inferolateral triangle is delimited anteriorly; by a line between the entry point of the trochlear nerve (CN IV) to the dural entry point of the abducens nerve (CN VI) at Dorello’s canal. Posteriorly, it’s bordered by a line between the dural entry point CN VI at Dorello’s canal and the superior petrosal vein at the superior petrosal sinus. Its superior border is the line drawn between the entry point of CN IV at the tentorium and the superior petrosal vein at the superior petrosal sinus

The infero-medial triangle is one of two paraclival triangles of the skull base. It is delimited medially by a line from the posterior clinoid process to the dural entry of the abducens nerve, laterally by a line from the posterior clinoid process to the dural entry of the trochlear nerve, and a base by a line from the dural entry of the abducens nerve and the trochlear nerve. Its contents are the posterior genu of the internal carotid artery and the dorsal meningeal artery [5, 6, 14, 32] (Figs. 4, 12) (Table 1).

### Inferolateral paraclival triangle

The inferolateral triangle consists anteriorly of a line from the abducens nerve's dural entry and the trochlear nerve's dural entry, laterally with a line from the entrance of the trochlear nerve and the petrosal vein, posteriorly with a line from the dural entry of the abducens nerve to the petrosal vein. Its contents are the porus trigeminus [5, 6, 14, 18] (Figs. 4, 12) (Table 1).

## Discussion

Claudius Galen (119–199 a.d.), a confidant of royalty and physician to the gladiators, dissected animals and quietly transposed his findings to human anatomy. Those animals had parasellar carotid retia bathed in venous blood, which humans do not have. Winslow took it upon himself to name it ‘‘cavernous sinus’’ (CS), two sinus cavernosi, one on each side, two orbitary sinuses, one on each side, and all these sinuses communicate with each other, and with the great lateral sinuses [23].

He thought that it would resemble the corpus cavernosum of the penis, which, in turn, he imagined to be a large, single, trabeculated venous cavern. His presumed concept of a single, large, trabeculated venous cavern persists today, becoming the most extended enduring myth in medical science [23].

Wepfer, in 1658, described the intra-cavernous internal carotid artery as passing through deep and conspicuous space [31].

Dorland, in 1985, found in a case of a long-standing arteriovenous fistula that the engorged and thickened ‘‘arterialized’’ veins were readily noted to be neither cavernous nor a dural sinus but a plexus of veins [23].

Schafer and Thane, in 1849; Anson, in 1953; Ferner, in 1963; Netter, in 1953, with beautiful drawings, they depict a plexus and call it CS, Anson in 1953 drew a single channel and called it a “plexus,” Spalteholtz, in 1938, drew a plexus with extension along the carotid canal on one page and a single large cavern on another page both labeled CS, Ferner, in 1963 drew a plexus with the actual extensions and labeled it CS [23].

Hamby, in 1966; Knosp et al., in 1987; Parkinson, in 1972; Taptas, in 1949, called the term CS inappropriate [23].

In 1965, Parkinson, the first deviser of the triangular space around CS, described the triangle between the trochlear and ophthalmic nerves to safely approach a lesion located at the internal carotid artery. Since Parkinson, several studies by clinical anatomists and neurosurgeons devised the triangular spaces around the CS. Since his pioneering studies, several critical triangular relationships formed by the convergence and divergence of cranial nerves have been described in the CS, in the middle cranial fossa, and in the paraclival region. Parkinson proposed the replacement of the ‘‘parasellar plexus of veins’’ in the ‘‘lateral sellar compartment’’ with the “parasellar plexus” because the plexus is present from early fetal life onward (Knosp et al., 1987; Solasol et al., 1966) extends about the sella in front of, behind, and beneath the pituitary beyond the lateral sellar compartments [22].

Browder and Parkinson performed the first cavernous sinus approaches to treat carotid-cavernous fistula. [2]

Parkinson, Dolenc, Taptas, and Umansky were pioneers in describing the surgical entry points into the sinus as triangular corridors. This geometric construct has been adopted as nomenclature for the region by most neurosurgeons [27].

Currently, cavernous sinus approaches are performed for basilar tip aneurysms, carotid-ophthalmic aneurysms, pituitary adenomas, some trigeminal neuromas, and other tumors in the region [20].

Although the anatomy of the cavernous sinus has been well described, the sinus remains a challenging and unfamiliar place for many neurosurgeons.

## Conclusion

Concise knowledge of the ten triangles is a strict requirement for any remarkable neurosurgeon. New surgical trans-triangle techniques or access pathways could be used to board different pathologies. We have left out measurements of each triangle to evade the premise of this simplified study. To thoroughly study each triangle profoundly, we advise you to investigate specific publications that only concentrate on each triangle or group of triangles.

## Data Availability

All data generated or analysed during this study are included in this published article.

## Abbreviations

CS: Cavernous sinus
GSPN: Greater superior petrosal nerve
ICA: Internal carotid artery
IAC: Internal auditory canal
